# “Please Don’t Send Us Spam!” A Participative, Theory-Based Methodology for Developing an mHealth Intervention

**DOI:** 10.2196/mhealth.6041

**Published:** 2016-08-17

**Authors:** Yoesrie Toefy, Donald Skinner, Sarah Thomsen

**Affiliations:** ^1^ Research on Community and Health Department of Community Health Sciences, Faculty of Medicine Stellenbosch University Cape Town South Africa; ^2^ Karolinska Institutet Department of Global Health Karolinska Institutet Stockholm Sweden

**Keywords:** mHealth, male circumcision, postoperative wound-healing period, health promotion, audio messaging, behaviour change

## Abstract

**Background:**

Mobile health solutions have the potential of reducing burdens on health systems and empowering patients with important information. However, there is a lack of theory-based mHealth interventions.

**Objective:**

The purpose of our study was to develop a participative, theory-based, mobile phone, audio messaging intervention attractive to recently circumcised men at voluntary medical male circumcision (VMMC) clinics in the Cape Town area in South Africa. We aimed to shift some of the tasks related to postoperative counselling on wound management and goal setting on safe sex. We place an emphasis on describing the full method of message generation to allow for replication.

**Methods:**

We developed an mHealth intervention using a staggered qualitative methodology: (1) focus group discussions with 52 recently circumcised men and their partners to develop initial voice messages they felt were relevant and appropriate, (2) thematic analysis and expert consultation to select the final messages for pilot testing, and (3) cognitive interviews with 12 recent VMMC patients to judge message comprehension and rank the messages. Message content and phasing were guided by the theory of planned behavior and the health action process approach.

**Results:**

Patients and their partners came up with 245 messages they thought would help men during the wound-healing period. Thematic analysis revealed 42 different themes. Expert review and cognitive interviews with more patients resulted in 42 messages with a clear division in terms of needs and expectations between the initial wound-healing recovery phase (weeks 1–3) and the adjustment phase (weeks 4–6). Discussions with patients also revealed potential barriers to voice messaging, such as lack of technical knowledge of mobile phones and concerns about the invasive nature of the intervention. Patients’ own suggested messages confirmed Ajzen’s theory of planned behavior that if a health promotion intervention can build trust and be relevant to the recipient’s needs in the first contacts, then the same recipients will perceive subsequent motivational messages more favorably. The health action process approach was also a useful tool for guiding the phasing of the messages. Participants were more positive and salutogenic than public health experts.

**Conclusions:**

The system showed how a process of consultation can work with a set of potential recipients of an mHealth service to ensure that their needs are included. Classic behavioral theories can and should be used to design modern mHealth interventions. We also believe that patients are the best source of messaging, ensuring that messages are culturally relevant and interesting to the recipient.

## Introduction

Voluntary medical male circumcision (VMMC) has been shown to reduce the risk of male acquisition of human immunodeficiency virus (HIV) by as much as 60% [1-3]. This led the World Health Organization/Joint United Nations Programme on HIV and AIDS to endorse that male circumcision should be promoted as an additional strategy in the prevention of HIV in men [4] and subsequently to plan for scale-up of VMMC services in 13 countries in Eastern and Southern Africa with high HIV prevalence and low rates of male circumcision, including South Africa [5]. However, there have been concerns about the quality of health education provided to men who are circumcised, particularly in relation to “risk compensation,” early resumption of sex, and the potential risk to their female partners [6]. Early resumption of sex, defined as having sex before the wound has healed, usually around 6 weeks, is a particular risk of widespread rollout of VMMC. Although challenges persist, including inadequate counselling, education, and follow-up, especially for married, discordant couples [7,8], very few programs have addressed early resumption of sex. VMMC counselling to encourage men to stay sexually safe needs to be maintained throughout this wound-healing period and to take into account the real-life risk factors of the circumcised men. Many overburdened public health systems are unable to provide adequate intervention programs to assist these recently circumcised men.

Recommendations have been made for developing and evaluating optimal counselling strategies among men seeking VMMC and to assess the effectiveness of behavior change communication strategies [9]. However, lack of human resources in the VMMC scale-up countries presents a barrier to the provision of such intense services, particularly if repeated contacts and encouragement are to occur. Moving programs to the realm of self-care would alleviate this burden and provide patients with high-quality care. Evidence from Southern and Eastern Africa indicates that some men, particularly those who are married or cohabiting, initiate sexual intercourse before the wound has completely healed. Identifying culturally acceptable and effective interventions is imperative to ensure that scale-up of VMMC in Eastern and Southern Africa is not accompanied by additional sexual morbidity and mortality. Furthermore, given the human resource shortages accompanying the rollout of VMMC in Eastern and Southern Africa, innovative strategies are needed for delivering these interventions. This is particularly the case when countries turn to independent partners to carry out mass, one-off VMMC campaigns outside of the normal constellation of services [10]. Clearly, more innovative strategies for communicating with, and effectively altering behavior in, men and their partners are needed. Further, given evidence of greater risk for early resumption of sexual activity in married or cohabiting men, strategies for navigating the postoperative period through, for example, nonpenetrative sexual activity should be developed and included in such education.

Self-care programs within the public health sector have been considered as a viable stopgap measure to address the service delivery challenges within the sector. Chronic staff shortages and budgetary constraints are common features of public health systems of most developing countries [11]. Due to these challenges, mHealth—the use of mobile phone technology to deliver health care—has emerged as an important and appreciated complement to health care education delivered through traditional channels [12]. Such technology may include the use of text messaging, video messaging, voice calling, and Internet connectivity. The potential for mHealth interventions to partially compensate for physical clinics in resource-poor areas is enormous [13].

Mobile health solutions have the potential of reducing burdens on health systems and empowering patients with important information. However, theory-based mHealth interventions are lacking, despite the fact that some theories of behavior change are well validated and tested on evidence-based interventions of prevention, diagnoses, and care [14-16]. Instead, all too often, mHealth interventions rely on the novelty of their modus operandi, as opposed to behavior change theory.

### South African Context

South Africa holds the dubious title of being the country with the highest number of HIV-positive individuals—over 5 million [17]. Based on the 2010 antenatal data, it was estimated that 18.5% of women in the Western Cape presenting at antenatal services were HIV-positive, with an incidence estimate of 6.2% [18]. The national prevalence based on these data was 30.2%. Transmission of HIV in South Africa is almost exclusively through heterosexual sex, thus heightening the importance of circumcision as a form of prevention. The South African Department of Health has made a commitment to rolling out medical circumcision in all provinces as one source of protection from HIV [4]. Approximately 5 million adult men could potentially be targeted. Circumcision is provided as part of a comprehensive service at district hospitals and includes HIV testing, counselling, and HIV education before the procedure.

Mobile phone use and access in South Africa is virtually universal. Mobile phone technology has been found to be acceptable and feasible for HIV- and AIDS-related prevention and services [19], and is now used in several health-related text-reminder projects in South Africa [20].

### Theoretical Frameworks Considered for the Development of Our Intervention

We found a scarcity of mHealth interventions that used change theory as the basis of the content development of the intervention. In fact, Tomlinson et al [21] maintained that nearly all mHealth intervention programs that are based on information transfer only, rather than an intervention that was designed through an iterative dynamic interaction between the system and the user, are more likely to fail at their implementation phase. Throughout the development of the intervention, we considered several theoretical frameworks for incorporation at different points in the intervention.

#### The Theory of Planned Behavior

The theory of planned behavior drives many behavior change interventions in existence today [22]. This theory evolved from the theory of reasoned action, which was proposed by Fishbein together with Ajzen in 1980 [23]. We used the three principle influencing factors proposed by Ajzen [14] of attitude toward the behavior, subjective norm, and perceived behavioral control to guide message selection and focus. For example, in our messaging development, the first norm, behavioral attitude, convinced the participants of the usefulness, worth, and advantages of the proposed behavior.

Within the mHealth domain, Ajzen and Fishbein’s theory of reasoned action has also influenced many modern technology acceptance models, such as Davis’s technology acceptance model [24,25] and Venkatesh and colleague’s unified theory of acceptance and use of technology [26]. These models replaced many of the attitude measures of the theory of reasoned action with the two technology acceptance measures, which are ease of use and usefulness.

#### The Action Research Approach

Jacobs and Graham [15] reviewed iterative health behavior intervention development and research methodologies and concluded that the requirements and solutions of these methodologies were evolved through collaboration between the developers and their intended target audiences. They promoted adaptive planning, and evolutionary development and delivery, and encouraged rapid and flexible response to change. This strategy minimizes the overall risk and allows a project to adapt to changes quickly [15]. It further contributes to the participants having an investment in and relating to the messages and intervention that is developed.

#### The Health Action Process Approach

The health action process approach (HAPA) proposes a sequence of two continuous self-regulatory processes, a goal-setting phase (motivation) and a goal-pursuit phase (volition). The goal-pursuit phase might be further subdivided into a planning phase, action phase, and maintenance phase. It is claimed that perceived self-efficacy plays a crucial role at all stages, along with other cognitions. For example, risk perceptions serve predominantly to set the stage for a contemplation process early in the motivation phase but do not extend beyond it. Similarly, outcome expectancies are chiefly important in the motivation phase when individuals balance the pros and cons of certain consequences of behaviors, but these expectancies lose their predictive power after a personal decision has been made. However, if one does not believe in one’s capability to perform a desired action, one will not adopt, initiate, and maintain it [16].

### Aims

To our knowledge, there are some interventions for promoting safe sex within a public health context, but none in the postoperative period after VMMC apart from sending simple text messages. Here we describe a participative, theory-based, mobile phone, audio messaging intervention attractive to recently circumcised men at VMMC clinics in the Cape Town area in South Africa. We designed the intervention to shift some of the tasks related to postoperative counselling on wound management and goal setting on safe sex. We place the emphasis on describing the full methodology to allow for replication of the method in other health care and prevention areas.

## Methods

We conducted this study as part of the formative phase of a clinic-based randomized controlled trial to evaluate the effectiveness of an automated telephone message system on reducing early resumption of penetrative sex among recent recipients of VMMC. The effectiveness of the intervention will be evaluated using a 2-armed, randomized, single-blind, controlled design, where the control group will receive the standard of care and the experimental group will receive the standard of care plus the study’s voice message intervention, described below, during the 6-week recuperation period [27].

### Setting

The study was conducted in catchment areas of the Heideveld Public Health Clinic and Mitchells Plain Hospital in Cape Town, in the Western Cape Province of South Africa. The study sites were chosen with the help of the provincial health department. The population demographics of these two areas are almost exclusively Coloured and Afrikaans speaking. The term Coloured refers to an official South African race group used in research and census data that is predominantly mixed ancestry. The term originated in the apartheid era but remains an important descriptor and label for a distinct community. More than 48% of the people who live in the Western Cape are classified as Coloured, mostly still living in defined communities that are at least 90% Coloured. Both areas are densely populated, with a low socioeconomic base and an average population density of 9600/km^2^. The housing is typically 1- to 2-bedroom maisonette housing. In 2011, the population of the suburb Mitchells Plain was 310,485 and the average household size was 4.57 people per household. There is a large Muslim population (10% to 15%) in the catchment area for one of the townships [28]. The Coloured community has a growing HIV prevalence rate—7.6% according to the latest antenatal data, indicating a generalized epidemic in this population [29]. The heightened HIV risk to this population group lies in a high illicit drug and alcohol use in the community, which is associated with risky sexual behavior [30].

### Design

**Figure 1:**
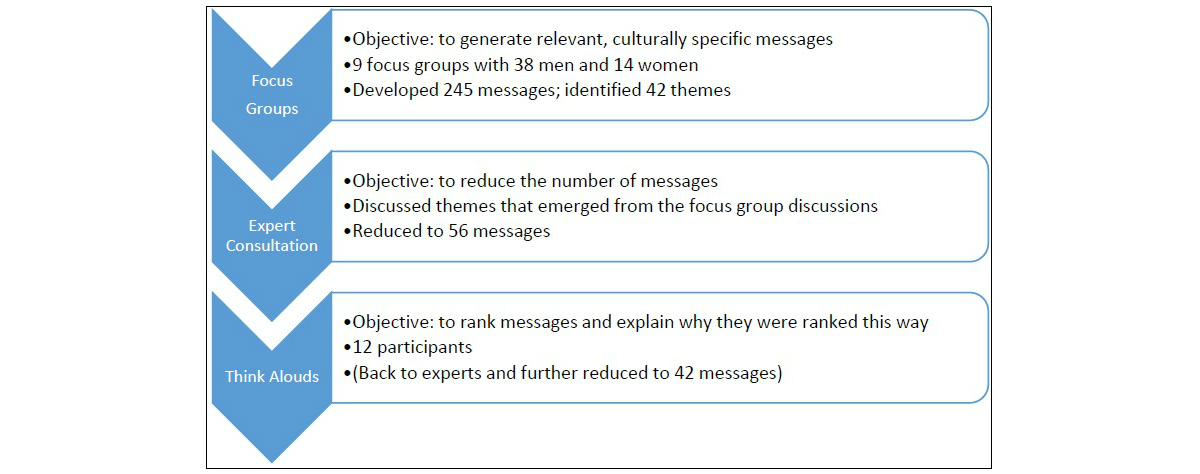
Steps to develop an mHealth intervention for recently circumcised men at voluntary medical male circumcision clinics in South Africa.

We used several participative, qualitative methods to develop mHealth phone messages and their sequencing, placing the user’s needs and experiences in the center. These were (1) focus group discussions with 52 recently circumcised men and their partners to develop initial messages they felt were relevant and appropriate, (2) thematic analysis and expert consultation to select final messages for pilot testing, and (3) cognitive interviews with 12 recent VMMC patients to judge message comprehension and to rank the messages (Figure 1). All interviews were conducted in Afrikaans, tape recorded, and then transcribed and translated into English.

Message content and phasing was guided by the theory of planned behavior and HAPA. The methods and incorporation of the theoretical frameworks are described in more detail below.

#### Focus Groups

In order to generate messages that would be considered appropriate and relevant for patients, we conducted 9 focus groups with a total of 52 men and women (6 male and 3 female focus groups) (Table 1). The methods of recruitment, conduct, and analysis of the focus groups are explained elsewhere [31].

**Table 1:** The study participants of the focus groups by sex and religion.

Focus group no.	Sex	No. of participants	Age range (years)	Religion	
				Muslim	Christian
1	Male	6	19–36	4	2
2	Male	6	22–45	3	3
3	Male	6	23–63	4	2
4	Male	7	18–42	5	2
5	Male	6	20–39	4	2
6	Male	7	21–53	4	3
7	Female	4	22–41	2	2
8	Female	5	19–34	3	2
9	Female	5	25–52	3	2

In addition to providing valuable information about the perceptions and behavior patterns of couples during the wound-healing period [31], participants of these focus groups were asked to write down 5 messages they felt could have assisted them during this period. The facilitators gathered these lists before the group discussions began and the researcher captured and translated the messages. We cleaned this list and identified common themes through use of thematic analysis using Atlas.ti version 7 (Scientific Software Development GmbH). Additionally, we collected information on the general acceptability of mobile messaging, generation of appropriate and relevant messages to recipients of VMMC during their 6-week recovery period, and the acceptable frequency of mobile messages. By getting the respondents to develop the theme list, we were drawing out the relevant issues to improve motivation and reduce blocks to volition, as well as identifying key behavioral, normative, and control beliefs.

#### Expert Consultation

The second part of this phase was to solicit expert opinion on the internal reliability and the contextual and cultural applicability of the messages. We consulted with a health communication expert from the South African Department of Health with extensive expertise in safe sex and behavior change, and local knowledge of the population and local conditions. As a first step, we grouped the messages into themes and then deleted incorrect, duplicate, and repetitive messages. Through interrater agreement, we decided on 1 or 2 examples representing each theme. We also adjusted several messages to reflect technical correctness and missing themes such as HIV prevention messaging. This decision was guided by existing theoretical models of behavior change, including the HAPA model. The order and frequency of the messages was guided by the recommendations of the focus groups and expert opinion. This phase is essential to ensure the accuracy of the messages and their constructive interaction with the health services.

#### Cognitive Interviews

The third phase of the message development was the validation phase, where we conducted cognitive interviews (“Think Alouds”) with 12 patients to rate the final selection of messages. We recruited a convenience sample of 12 recently circumcised men ages 23–39 years (mean 29.6 years) from the Heideveld and Mitchells Plain clinics to participate in this part of the study. Of these, 5 were Muslim and the rest were Christian. All were Coloured except for 2, who were foreign nationals staying in the area. We recruited men who had completed their 6-week wound-healing period during the previous month. The men were recruited from the 2 clinics’ circumcision intake registries. They were recruited via telephone, and the researcher subsequently interviewed them at convenient places in the community.

We asked the participants to evaluate and rank messages based on their appropriateness and effectiveness in the 42-day wound-healing period. The messages were put in groups of 3–6 messages for ranking purposes. Each message was read out to the participants and then tested for comprehension, such as having the participants repeat the message in their own words. We then probed for the participants’ perception of the aim of each message.

Following each section, we asked participants to reflect on the time period in the 42-day period for which we had designed this group of messages. They were then asked to rank the messages in the group for appropriateness and clarity. Following the ranking exercise, we asked participants, using a think-aloud method, to reflect on message grouping and whether there were any messages missing that could have made a difference, on whether there were any unnecessary and inappropriate messages, and on the reasons why they ranked the group in a particular way. This refined the contributions from step 1, improving the messages in terms of both the theory of reasoned action and HAPA.

## Results

### Step 1: Generation of Messages and Key Information on the Messaging

We had two goals for this stage: the first one was to get an unprompted set of messages from the recently circumcised men and their partners. We ended up with 245 messages the participants felt would have had an impact on their behavior and attitudes during the 42-day postoperative wound-healing period. The second aim was to solicit group responses to questions around frequency, nature, and order of the messages.

Focus group participants’ suggested messages covered 30 different themes. Some examples were wound and pain management, the roles of rest and family support, erection issues, how to stay healthy, when to resume sex, alcohol use, affirmation, sexual needs of partners, and condom use. Themes could be further grouped into phases and practical versus motivational messages. The latter aspects are described more fully below, since they have an impact on how the intervention was later designed. The selection of messages covered the dominant areas of the HAPA theory—that is, planning and its components of self-efficacy, goal setting, and action.

#### The 2-Phase Approach

Participants in all focus groups repeatedly stressed that the 42-day period following the circumcision surgical procedure is roughly divided into a wound and pain management period (the first 3 weeks) followed by the adjustment period. The first phase is characterized by practical issues such as wound and pain management, while the second phase is dominated by motivational and planning issues. Examples of comments from the focus groups are

[First phase:] I think for the first period, it should be about pain management and how to take care of oneself.

Male, Muslim, 27 years

[Second phase:] Later, anything to encourage me to stay safe would be nice.

Male, Christian, 32 years

Examples of participants’ suggested messages in the 2 different phases are

[First phase:] Rest and don’t do anything strenuous in the first week.

Female, Muslim, 21 years

[Second phase:] You did something great. Be proud of yourself.

Male, Muslim, 51 years

#### Practical Versus Motivational Messaging

Participants also clearly distinguished between 2 different types of messages—practical and motivational—which are also associated with the 2 phases. Thus, *in the first phase*, practical messages are linked to coping themes such as inactivity and rest, wound healing, role of the clinic, and support mechanisms:

[Inactivity and rest:] Rest! This will help with the wound healing.

Male, Christian, 24 years

[Wound healing:] Don’t mess around with the wound. No home remedies!!

Male, Muslim, 32 years

[Role of the clinic and medication:] If there’s any problem down there, go to the clinic to fix it.

Female, Muslim, 28 years

[Support mechanisms:] Are you having a difficult time? Talk to someone you love. They will understand.

Male, Christian, 35 years

Motivational messages, however, which were associated with *the second phase*, were closely linked to themes of safe living, the needs of the partner. and goal setting:

[Safe living:] I think the messages should be about safe living and making sure you take responsibility for your actions.

Male, Christian, 45 years

[Needs of partner:] Take your time when you are pleasing your partner.

Female, Muslim, 30 years

[Goal setting:] No sex or masturbation for six weeks![Male, Muslim, 21 years];Want to be healthy? Look after yourself.

Male, Christian, 47 years

#### Salutogenic Nature of the Messages

An interesting aspect of the focus groups was the emphasis on positive and inspirational messages as opposed to messages that emphasized disease and negative consequences:

I would like some inspirational messages.

Male, Christian, 54 years

Start every day with a good attitude.

Female, Muslim, 24 years

Be proud of yourself. You did it!

Male, Muslim, 30 years

Thus, the majority of the messages that former patients and their partners suggested were positive and assertive.

#### Frequency of Messages

In addition to providing message content, participants were asked about their opinions on how often messages could be sent to them.

Participants were wary of unsolicited and invasive messages:

If they get too many messages, even if they are good, people won’t listen.

Male, Muslim, 43 years

This (the frequency of the messages) is very difficult to calculate. I don’t think you want to turn this into spam.

Female, Muslim, 25 years

The groups were asked of ways to counter this natural resistance to “push” messaging campaigns. The first strategy given was prior consent:

I guess if I sign up for it and expect it, then it should be okay.

Male, Muslim, 20 years

The other strategy was a clear end-date of the program:

I don’t think men would mind getting messages because they know it’s only for a certain period.

Male, Christian, 31 years

### Step 2: Expert Consultation

In consultation with a health communication expert from the South African Department of Health, we reduced 245 messages to 56. This reduction was done by removing identical or similarly structured messages, and amalgamating messages of a similar theme under one exemplar. An example of this was that the messages “Have lots of rest. It will help with your recovery,” “Rest and sleep. This will help with the recovery,” “Sleep a lot. It will help with the healing,” and “Resting will help with the wound healing” were amalgamated into “Rest! This will help with the wound healing.” We made these decisions based on extensive local knowledge of the population and local conditions, and safe sex and behavior change. Table 2 shows the full set of resultant themes by phase once all the messages were combined into a reduced set of thematic constructs, while remaining responsive to the community’s felt needs. One message is provided for each theme as an example.

**Table 2:** Themes and example messages by phase after voluntary medical male circumcision resulting from expert consultation.

Phase^a^	Themes	Examples of messages
1	Inactivity and rest	*Rest! This will help with the wound healing.*
1	Role of the clinic; wound management	*Go back to the clinic after two and seven days so the dressing can be replaced and the wound can be checked.*
1	Wound management; hygiene	*Use lukewarm water to wash the wound every day. Keep the dressing dry.*
1	Wound management; erection issues	*Check for any skin tightness when you get an erection.*
1	Role of the clinic; wound management	*If there is pus coming out of the wound, go to the clinic.*
1	Pain management; medication	*Don’t be brave! Take pain tablets to relieve the pain.*
1	Wound management; partner and family support	*Are you having a difficult time? Talk to someone you love. They will understand.*
2	Self-efficacy and affirmation; goal setting	*Want to be healthy? Look after yourself.*
2	Self-efficacy and affirmation	*You can do it!*
2	Partner and family support	*Make sure you talk to your partner.*
2	Self-efficacy and affirmation; motivation	*Did you look after your penis today?*
2	Goal setting; healthy living	*Regular exercise and healthy diet are essential.*
2	Wound management; erection issues	*Get rid of that painful erection by urinating frequently.*
2	Early resumption of sex	*Having sex too early will just set you back!*
2	Early resumption of sex; alcohol consumption	*Be aware of alcohol. It impedes your judgement.*
2	Safe living; condom use	*Remember that circumcision does not provide 100% protection.*
2	Motivation; goal setting	*No sex or masturbation for six weeks!*
2	Nonpenetrative sex; sexual needs of partner	*Find ways to please your partner without using your penis.*
2	Nonpenetrative sex	*Loving is not about sex only!*
2	Self-efficacy and affirmation; motivation	*You are the best!*
2	Motivation; goal setting	*Regular condom use, knowing your HIV* ^b^ *status, and keeping to one partner is the recipe for an HIV-free future.*

^a^Phase 1: recovery phase; phase 2: adjustment phase.

^b^HIV: human immunodeficiency virus.

### Step 3: Cognitive Interviews

Think-aloud participants engaged intimately with the messages and generally confirmed the set of themes proposed by the previous step. Their interaction with the messages originated from their own experiences during the wound-healing period and confirmed the elements of motivation and volition as proposed by the HAPA model. Although we previously divided the 6-week period into the initial wound-healing recovery phase during weeks 1 to 3 and the adjustment phase from weeks 4 to 6, we now separated the 6-week period into three 2-week periods for ease of discussion. Some of the comments and recommendations made on each 2-week period of the 42-day recovery period follow.

#### Messages for Weeks 1 and 2

This period was dominated by pain and wound management themes, as the participants ranked messages such as “Rest! This will help with the wound healing” and “Do not pull or scratch the wound while it is healing” the highest in their groupings. Toward the end of this period, the patient’s support structure was also given priority: “Are you having a difficult time? Talk to someone you love. They will understand.” The group also recommended that the frequency of phone messages to patients should be twice a day at the beginning of the period, tapering to once a day after the first 2 days, to provide additional support for the days following the operation.

Messages that scored the lowest among the participants were those that the group found impractical, such as “Check for any skin tightness when you get an erection.” In essence, expert opinion dictates that this is an important first visual check of whether the stitching is not impeding or pulling the skin during erections, and that it should be done as early as possible so any corrective surgery can be done before the wound area heals completely. Participants found this expert-inserted message problematic because, during the preoperative wound-care talk, strong emphasis was placed on the danger of prolonged erection and the tearing of the stitches. Further, patients were instructed how to get rid of erections as soon as they could. Patients found their first erection too stressful to pay proper heed to the message’s instruction. Another message that scored very low during this period was the inspirational message “You can do it.” Participants generally liked the intent of the message but felt that it should come later, and they were more interested in practical advice on how to get through this period.

#### Messages for Weeks 3 and 4

The messages in this period reflect the changing priorities of the patient. The wound is practically healed and the patient is going through a period of how to adjust to this new feature, not only sexually, but also aesthetically and the way it feels. Messages with recovery themes, such as “Involve your partner in your recovery period” and “Get rid of that painful erection by urinating frequently,” still scored high, but messages such as “Regular exercise and healthy diet are essential,” reflecting adjustment themes, also received high rankings. Expert-driven messages, once again, scored low. Messages such as “If you have sex before the wound is properly healed, there is a greater chance of contracting STIs [sexually transmitted infections] or HIV” were looked at as too academic and preachy. Motivational messages that were too generic, such as “You did it!,” were scored lower than more practically orientated motivational messages such as “Think about what you are going to do before you do it.” Messages on avoiding penetrative sex were also well received during this period.

#### Messages for Weeks 5 and 6

The high-scoring messages in this period revolved around alternative sexual activity, such as “Loving is not about sex only!” and “Take your time when you are pleasing your partner;” validation, such as “You lost the skin! Can you feel the difference?;” and looking-ahead messages, such as “You are planning for your future.” Low-scoring messages, as in the previous period, were those expert-adjusted messages that were perceived as artificially instructive, such as “Regular condom use, knowing your HIV status, and keeping to one partner is the recipe for an HIV-free future.”

## Discussion

We developed a formative research process to develop theory-driven and contextually based mHealth messages to provide additional counselling and support to recently circumcised men during their wound-healing period. We employed a process where the targeted group created the initial messages and then the targeted group, researchers, and communication and health experts collaboratively refined and optimized the message regime for the intended period.

This study’s findings suggest that phone messages should be relevant, positive, simple, few in number, and designed to be contextually appropriate for the intended audience. In order to inform and motivate these men, messages must address the reality of the experiences the men are facing throughout the 6-week period. One method is to directly engage men and their partners in iterative discussions about message design and delivery, and involve them in the development of messages so that messages are relevant and meaningful for the recipients.

A few studies have explored the construction and development of health messages delivered through a mobile device using a multiphased approach with a strong target group participation. Ybarra et al [32], in their aim to develop an mHealth HIV prevention program for adolescent gay, bisexual, and queer men, took the message development through 5 iterations with target audiences contacted in the first phase to test acceptability of the messages and during the last phase with beta testing. They found, as in our study, that the men preferred positive and friendly content that, at the same time, did not try to sound like a peer [32]. Similarly, Woolford and colleagues [33] explored obese adolescent participants’ perspectives related to weight management messages, and they found enthusiasm for short message service text messages as a strategy to support weight loss efforts among these participants. Importantly, the findings from their study also support our study in that teen participants desired brief, positive, encouraging messages that had a “natural” tone and made specific reference to the teen demographic [33]. Another mHealth weight management program conducted by Hingle and colleagues in 2013 using a multistage youth-participatory approach found similar results [34]. The main difference between our study and the ones mentioned above is that the source of the messages in our study was the target audience and not researchers and experts.

A few studies have used behavioral change theory in the development of messages. Hingle et al [34] developed an mHealth intervention to influence the knowledge, attitudes, and behavior of adolescents on nutrition and physical activity. They used a 3-phased youth-participatory approach to develop and test messages. The first phase was content identification and initial message development, the second was message testing and refinement, and the last phase was pilot testing of a message delivery protocol [34]. Other studies by Bock et al [35] and Ybarra et al [32] also concluded that partnering with the target population in the message development is critical to ensure that a salient final product and feasible protocol are created. The pathologic versus the salutogenic nature of the messages has also been shown as a factor in the rate of acceptance of the mHealth interventions [36,37].

Our study used a participatory approach on two levels to take the needs and views of the target population into account in the development of the messages and the frequency of messages. The first level was on the generation of the messages, which was initiated by the target audience, followed by the reflection of expert opinion on these messages. The next iteration, with a cleaner and more theme-centered set of messages, was evaluated by a group of recently circumcised men for relevance and impact.

This approach recommended the development of a progression of the messages. The formative focus groups told us that the period of 42 days following the VMMC procedure is a series of progressive phases, from the purely physical pain and wound management in the early phase of healing, then to that of adjustment, to how their circumcised penis was looking and feeling, followed by an external adjustment to physical movement and sex drive. The content of the messages needed to reflect this continuous movement through the 42 days. So from more practical messages centered on pain and wound management, messages moved to more inspirational and “planning-ahead” messages.

In this study, we found that applying this theoretically informed approach carefully resulted in message content that was consistent across different recuperation phases. We identified certain key themes that we felt would be useful in the design of mHealth messaging content, as they may facilitate men’s healing rates and perceived self-efficacy to attend the clinic and other healing strategies, reduce or minimize potential fears, and provide an impetus for self-care. These included maximizing content relevancy in messaging, encouraging and validating the patient, and providing content that suggested that immediate support structures such as the man’s partner, family, or the health care workers were role players in their own recuperation.

It emerged that using the content suggestions of circumcised patients as the genesis of the program’s message development could help build on men’s already intuitive sense of healing strategies, their knowledge of the benefits of following clear recuperation methods, and what was acceptable to them. This mix of experience and the knowledge of the benefit of adhering to the healing regimen can be leveraged to get men back to the clinic at their appointed schedule and follow the recommended healing program, with the mobile messages functioning as cues to action.

There was an interesting dichotomy in the messages that experts considered as essential messaging for circumcised men in their wound-healing period, such as countering the perception that circumcision replaces condom use; or standard HIV prevention messages, with the circumcised men’s perceptions of what essential messages were. The expert messages scored consistently low in desirability or impact. It could be that the applicability aspect was low because most of the participants sought out the procedure for reasons other than HIV prevention, or it could be that there is an oversaturation of these messages and the low scores merely reflect a natural irritation caused by these “academic” messages. Related to this is the participants’ strongly held consensus that salutogenic and caring messages would provide a stimulus to trigger men to take the necessary action to heal properly and refrain from penetrative sexual encounters. The salutogenic model is useful for all fields of health care, especially in the field of health promotion, as it provides a direction and focus, allowing the program to move away from disease and risk factors and to focus on the salutary and seeing the entire person in relation to the disease [36].

### Implications for Research and Practice

This study demonstrates a novel way in which to engage men in conversations about health using a familiar and ubiquitous communication method. Rather than being passive recipients of top-down, expert-driven communications, participants in this study had the opportunity to actively participate in the message design process and engage with health information through informal interactions with experts and with one another, thereby increasing the likelihood that they would adopt the recommended behaviors. Based on our findings, this methodological approach to the development of theory-driven, evidence-based, and culturally appropriate health messages in mobile health interventions could be adapted to other cultural and geographic environments and various health issues. This approach can be used as a model to test and adapt health messages in a variety of mobile health intervention projects within a variety of cultural contexts. It can also be applied to health communication message development more generally.

Additional research is needed to determine whether this approach facilitates technology-based interventions to be an effective, sustainable way to promote healthy lifestyles to circumcised men and have a significant impact on behaviors that place men at increased risk. These messages are being evaluated in a randomized controlled trial among approximately 1200 men in 7 VMMC clinics in Cape Town, South Africa.

### Conclusion

mHealth has the potential to provide a valuable health care service in the context of providing an expanded VMMC in resource-restricted circumstances. To provide this service, we argue that the intervention platform, the message content, and the delivery timing have to be responsive to the felt needs of the respondents, as well as providing good factual information. As with other interventions, mHealth also needs to use established behavioral theories to develop and construct interventions and messages.

## Abbreviations

HAPA: health action process approach
HIV: human immunodeficiency virus
VMMC: voluntary medical male circumcision
